# Lanthanide-Doped Ceria Nanoparticles as Backside Coaters to Improve Silicon Solar Cell Efficiency

**DOI:** 10.3390/nano8060357

**Published:** 2018-05-23

**Authors:** Ali Hajjiah, Effat Samir, Nader Shehata, Mohamed Salah

**Affiliations:** 1 Electrical Engineering Department, College of Engineering and Petroleum, Kuwait University, Safat 13133, Kuwait; 2 Center of Smart Nanotechnology and Photonics (CSNP), SmartCI Research Center, Alexandria University, Alexandria 21544, Egypt; effat_samir@mena.vt.edu (E.S.); nader83@vt.edu (N.S.); n.shehata@kcst.edu.kw (M.S.); 3 Department of Electrical Engineering, Faculty of Engineering, Alexandria University, Alexandria 21544, Egypt; 4 Department of Engineering Mathematics and Physics, Faculty of Engineering, Alexandria University, Alexandria 21544, Egypt; 5 USTAR Bioinnovation Center, Faculty of Science, Utah State University, Logan, UT 84341, USA; 6 Kuwait College of Science and Technology (KCST), Doha Spur Rd., Safat 13133, Kuwait

**Keywords:** solar cells, ceria nanoparticles, lanthanide doped, conductivity, photoluminescence intensity

## Abstract

This paper introduces lanthanide-doped ceria nanoparticles as silicon solar cell back-side coaters, showing their influence on the solar cell efficiency. Ceria nanoparticles can be synthesized to have formed oxygen vacancies (O-vacancies), which are associated with converting cerium ions from the Ce^4+^ state ions to the Ce^3+^ ones. These O-vacancies follow the rule of improving silicon solar cell conductivity through a hopping mechanism. Besides, under near-ultra violet (near-UV) excitation, the reduced trivalent cerium Ce^3+^ ions are directly responsible for down converting the un-absorbed UV wavelengths to a resultant green photo-luminescence emission at ~520 nm, which is absorbed through the silicon solar cell’s active layer. Adding lanthanide elements such as Neodymium “Nd” as ceria nanoparticle dopants helps in forming extra oxygen vacancies (O-vacancies), followed by an increase in the number of Ce^4+^ to Ce^3+^ ion reductions, thus enhancing the conductivity and photoluminescence down conversion mechanisms. After introducing lanthanide-doped ceria nanoparticles on a silicon solar cell surface, a promising enhancement in the behavior of the solar cell current-voltage curve is observed, and the efficiency is improved by about 25% of its initial value due to the mutual impact of improving both electric conductivity and optical conversions.

## 1. Introduction

Silicon (Si) is considered as the dominant commercial material worldwide for solar cell production [1]. However, there are many optical and electrical issues that lead to overall efficiency reduction. One of the main optical problems is that Si solar cells can absorb only 55% of the incident irradiation photons spectra, but the remaining spectra is not used or absorbed, and is considered a loss. These un-absorbed incident photons may have energies less or more than that silicon bandgap can absorb [2,3]. Therefore, converting these low-photon energies to higher ones can improve the absorbed spectrum of solar cells and consequently enhance the efficiency of the cell [4,5]. Optical conversion processes are considered as the best ways to improve Si solar cell efficiency optically, by harvesting the non-absorbed infrared (IR) or ultraviolet (UV) solar spectrum through optical up-conversion or photoluminescence down-conversion mechanisms, respectively [6]. Moreover, there are some electrical issues such as the high resistivity of the metal contact and the formation of the parasitic resistance which can reduce the surface conductivity of the pathways of the generated photoelectrons. That can be considered another source of efficiency reduction of solar cells [7].

In this presented research work, both photoluminescence and conductive features of ceria nanoparticles are investigated as silicon solar cell surface coaters for efficiency improvements. Since, ceria nanoparticles are considered as down conversion nanostructures, they should be introduced onto the front side of the solar cells surface [8]. However, introducing down conversion materials onto the front side of solar cells may lead to some noticeable issues. These issues include the material’s effect on the absorbance of visible energy photons and the shadowing effect that may prevent sun photons from reaching the cell’s active layer [4,8]. Therefore, these challenges of introducing down conversion materials on the front side of the solar cells may not help in to improve efficiency. To avoid such issues, the ceria nanoparticles layer was investigated as a rear-side coater on the silicon solar cells. In this application, ceria nanoparticles may have a mutual impact of improving both electric conductivity and UV photon optical conversions. Despite the limited numbers of incident UV energy photons that might reach the ceria nanoparticles layer for the conversion mechanism, ceria nanoparticles conductivity might help with efficiency improvements. Formed oxygen vacancies inside ceria nanoparticles could help in improving solar cell conductivity through electron transitions to the external load through a hopping mechanism [9]. Doping ceria with some low activation energy lanthanides, for instance Neodymium “Nd” of different concentrations, could lead to a decrease in the bandgap of the un-doped ceria nanoparticles, which leads to Ce^3+^ ion reduction and an increase in the corresponding formed O-vacancies concentration and, hence, an increase in the material conductivity. Moreover, the high influence of the Ce^3+^ reduction, consequently enhances the down conversion of the UV photon energies to visible ones [10]. During this work, different electrical and optical characterization measurements such as conductivity, photoluminescence emission intensity, absorbance, and the allowed bandgap are investigated for both un-doped and Nd-doped ceria nanoparticles. These measurements are obtained to prove the existence of the formed charged O-vacancies, which are responsible for the mutual impact of improving both electric conductivity and optical down conversions of Si solar cells.

## 2. Materials and Methods

Chemical precipitation was selected for the synthesis procedure of ceria nanoparticles for its simplicity and the low cost of the required chemicals [10,11]. Cerium (III) chloride heptahydrate (99.9%, Sigma-Aldrich, St. Louis, MO, USA) of 0.5 g was dissolved in 40 mL of deionized water under constant stirring for 2 h in a 50 °C water bath. A few minutes after stirring began, 1.6 mL of ammonium hydroxide (28% NH_3_ in H_2_O, ≥99.99% trace metals basis, Sigma-Aldrich, St. Louis, MO, USA) was added as a catalyst. After the two-hour heating stage, the solvent was stirred at room temperature overnight. In the case of preparing neodymium-doped-ceria, neodymium (III) chloride (heptahydrate (99.9%, Sigma-Aldrich, St. Louis, MO, USA) was added to the initial precursor of cerium chloride and ammonium hydroxide according to the weight ratio of doping up to 10 wt. %.

Structural analysis of the synthesized nanoparticles was analyzed using a JEOL Transmission Electron Microscope (TEM) to check the mean diameter of nanoparticles along with Rigaku X-ray Diffraction (XRD) to confirm the structure peaks of ceria nanoparticles. Optical characterization measurements for both un-doped and Nd-doped ceria nanoparticles were obtained by UV-Visible-Near Infrared (UV-Vis-NIR) spectrometer (PG 92 spectroscopy, Lutterworth, England, UK), which was used to detect the absorbance dispersion so the direct bandgap could be calculated. Then, in order to measure the photoluminescence intensity for both un-doped and lanthanide-doped ceria nanoparticles, the experimental apparatus used was the same setup discussed in other of our related research papers such as [12]. In this setup, violet Light Emitting Diode (LED) with a centered wavelength of 430 nm is exposed to the synthesized nanoparticles solution and a monochromator is attached to the sample perpendicular to the excitation source for minimum scattering impact. Then, the optical output of the monochromator is detected by a photomultiplier tube (PMT, Newport 77360, Irvine, CA, USA) followed by a power meter (Newport, 1918R, Irvine, CA, USA) to record the optical visible fluorescent emission.

In this study, the main target was to improve Si solar cell efficiency using the two studied ceria nanoparticles; un-doped and lanthanide Nd-doped. During this work, commercial crystalline silicon solar cells with efficiency around 15% were used directly without any further preparation. The conductivity of the ceria solution was measured by Thermo Scientific Orion Star A329 (Pittsburgh, PA, USA) pH/ISE/Conductivity/RDO/Dissolved Oxygen Portable Meter. Ceria nanoparticle deposition onto the Si-solar cell surface was achieved by introducing a smooth thin layer of either un-doped or Nd-doped ceria nanoparticles onto the rear side of the purchased fragile Si solar cells. The technique used was a spin coating mechanism, which is considered as one of the simplest and fastest techniques to obtain smooth, thin layers compared to other coating techniques [13,14]. The spin coater (VTC-50A, Richmond, CA, USA) device was adjusted to accelerate up to a speed of 1500 rpm, and then was left at this rotation speed for 20 s during the deposition step. The spinning was done at room temperature. The mentioned parameters were optimized, thus, below these values, the achieved layer was rough and ceria nanoparticles agglomerated on the solar cell backside surface, while above these parameters, the used fragile Si solar cells were totally broken and smashed, especially when the speed of the spin coater increased. Finally, the full I-V characterization curves of both coated and un-coated solar cells were obtained using a PET-solar simulator (SS200ABA, Irvine, CA, USA).

## 3. Results

### 3.1. Synthesized Nanoparticle Characterization

**Figure 1:**
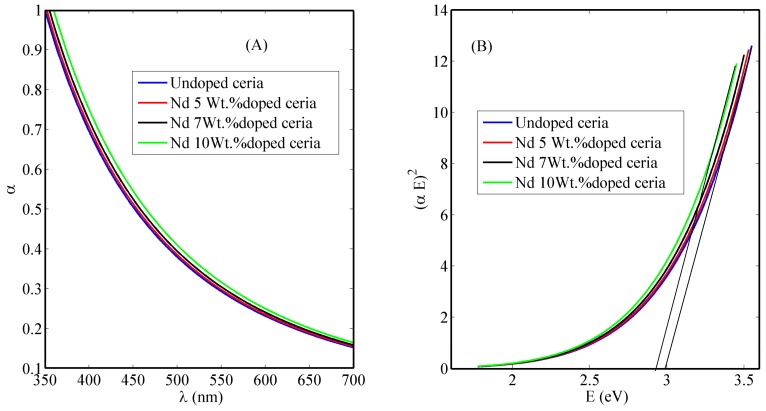
(**A**) Absorbance dispersion curves and (**B**) direct allowed bandgap calculations of un-doped, and Nd-doped ceria nanoparticles.

The optical absorption curves of un-doped and lanthanide Nd-doped ceria nanoparticles are shown in Figure 1A. From the absorbance curves, the direct allowed bandgap could be calculated from the linear region based on the following equation [15], which is shown in Figure 1B.

*α* (*E*) = *A*(*E − Eg*)^1/2^ (1)

where *α* is the measured absorbance coefficient, A is a constant that depends on the materials’ electrons and holes effective masses, *E* is the absorbed photon energy, and *Eg* is the calculated allowed direct bandgap. Experimentally, ceria nanoparticles’ accepted bandgap range is from 2.7 eV to 3.7 eV, depending on the synthesis method, temperatures, and size of the particles [16]. Direct allowed bandgap is a result of the reduction process during the synthesis process that converts Ce^4+^ ions to Ce^3+^ ions. The release of these reduced Ce^3+^ ions is associated with the formation of O-vacancies as discussed before. The calculated direct bandgap of our synthesized ceria nanoparticles is nearly 3 eV for un-doped ceria and is slightly less, up to 2.9 eV, with increased concentration of neodymium. That gives an indication that there are more formed free O-vacancies associated to more formed Ce^3+^ ionization states when the concentration of Nd is increased in the ceria nanoparticles, due to the relatively-low association energy between neodymium and vacancies [10].

**Figure 2:**
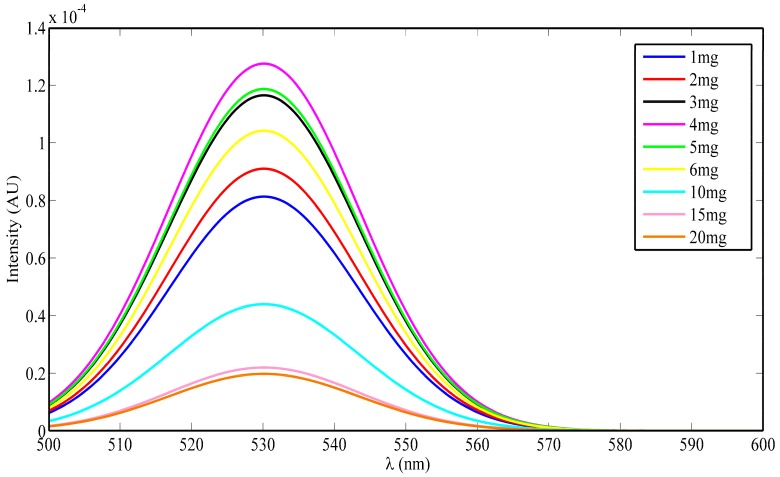
Photoluminescence emission spectrum of different un-doped ceria nanoparticle concentrations, per 3 mL distilled water solution.

Photoluminescence intensity measurements for different ceria nanoparticle concentrations are shown in Figure 2, and they were obtained to ensure the impact of the formed Ce^3+^ ions was the cause of the down-conversion process. Under near UV-excitation, the formation of optical visible emissions were centered at 520 nm, which corresponds to the formation of excited Ce^3+^ ions in Ce_2_O_3_ via the 5d-4f transition, and results in visible photon emissions. Therefore, higher concentration of Ce^3+^ states in CeO*_x_* with higher concentrations of the associated O-vacancies can lead to stronger photoluminescence emissions in neodymium dopant ceria, compared to un-doped ceria [17,18,19].

**Figure 3:**
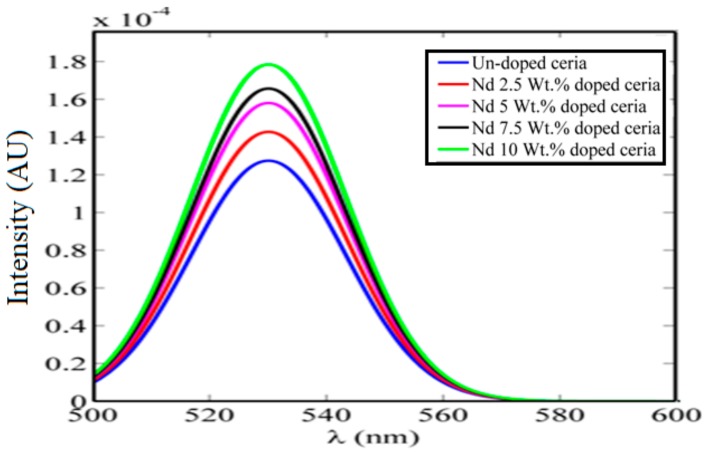
Photoluminescence emission spectrum of un-doped, and lanthanide Nd-doped ceria nanoparticles with different concentrations.

Generally, photoluminescence emission peaks of lanthanide-doped ceria nanoparticles are higher than those of the un-doped ceria, as shown in Figure 3. Generally, higher tri-valent cerium ions with associated O-vacancies are responsible for the visible emission of ceria according to the 5d-4f transition. The neodymium dopant increases the probability of having more tri-valent cerium ions with a higher probability of more O-vacancy formations with lower activation energy or with higher mobility [10,12,20]. The conductivity of the colloidal un-doped and lanthanide Nd-doped ceria nanoparticles was obtained using a Thermo Scientific Orion conductivity probe, as shown in Table 1.

**Figure 4:**
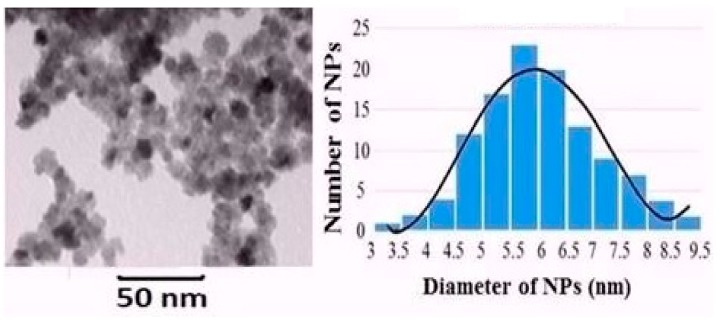
TEM image of ceria nanoparticles with mean diameter size of 6 nm.

**Figure 5:**
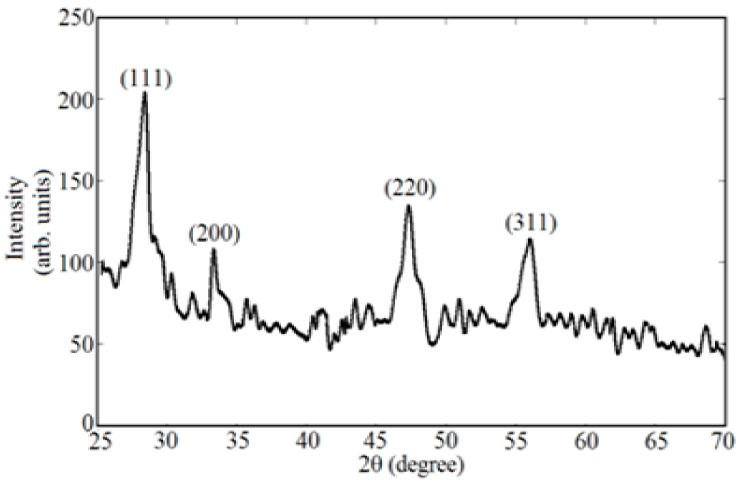
XRD pattern of Nd-doped-ceria nanoparticles.

**Figure 6:**
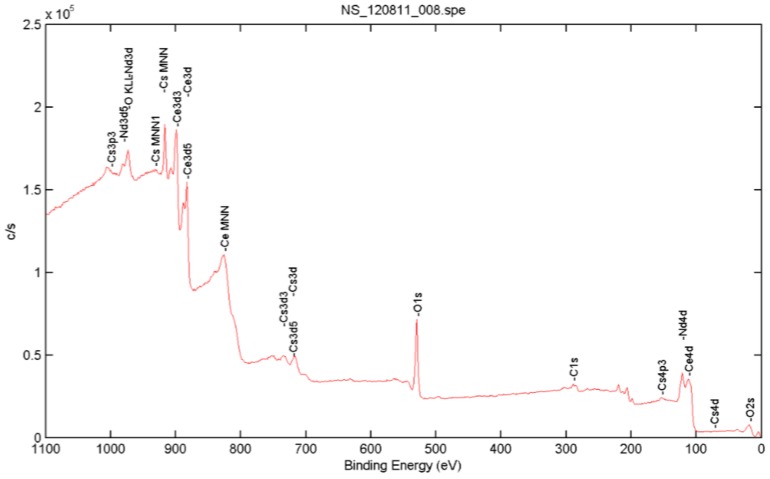
X-ray photoelectron spectroscopy (XPS) analysis of Nd-doped ceria nanoparticles.

Generally, lanthanide-doped ceria nanoparticle conductivity is higher than that of the un-doped ceria nanoparticles, due to the higher formed O-vacancies that improve the electron flow within the material through hopping mechanisms [12,20]. The mean diameter of the synthesized ceria nanoparticles was determined from TEM images and was found to be ~6 nm, as shown in Figure 4. The crystalline structure of the doped nanoparticles was analyzed using XRD, as presented in Figure 5. From the first diffraction peak of the most stable surface plane of ceria, (111) plane, the mean diameter was confirmed to be ~6 nm using Scherrer’s formula [21,22]. The XRD pattern is the same for both un-doped and Nd-doped ceria, which proves that there are no neodymium oxide patterns formed, and neodymium is only a dopant inside the crystalline structure of ceria [10]. From the XRD pattern of the most stable state of ceria, the lattice constant of ceria nanoparticles was calculated to be 5.22 A for un-doped ceria, and increased to 5.30 A and 5.35 A in the case of 5 wt. % and 10 wt. % of neodymium dopant, respectively. The X-ray photoelectron spectroscopy (XPS) analysis for Nd-doped ceria nanoparticles is presented in Figure 6, which shows the neodymium dopant exists inside the crystalline structure of ceria, and all the optical characteristic changes are due to the Nd-dopant.

### 3.2. Solar Cell Characterization

**Figure 7:**
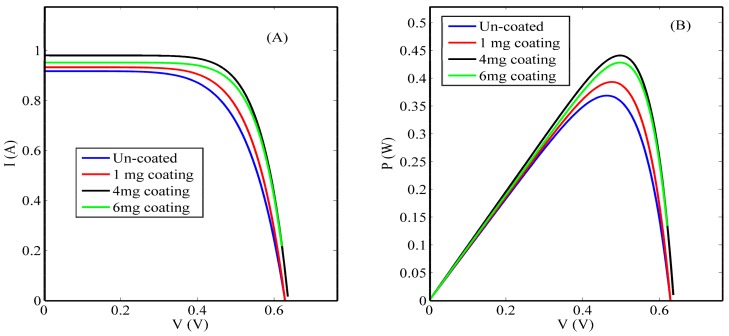
(**A**) I-V curves and (**B**) P-V curves of un-coated and coated cells with different un-doped ceria nanoparticle concentrations.

Figure 7 shows the influence of introducing different concentrations of the un-doped ceria nanoparticles upon the Si solar cells’ current-voltage (I-V) and power-voltage (P-V) curves. A detailed comparison between the un-coated and coated Si solar cells’ electrical parameters is presented in Table 2, which shows a promising improvement in the overall efficiency and some other solar cell electrical parameters. It is obvious that coating the rear surface of Si solar cells with 4 mg/mL concentrations of ceria nanoparticles shows the highest power efficiency conversion (PEC) improvement among the rest of the concentrations, which was also the highest obtained intensity in the photoluminescence emission spectra curve. Above this threshold concentration, reduction of fluorescence intensity emission is observed due to the optical quenching or scattering effect. The obtained improvement was from 14.74% to 17.64%, corresponding to about a 20% increase from the initial value. The calculated electrical parameters obtained from the measured I-V curves show the improvement of the short circuit current (Isc) due to the effect of the synthesized nanoparticle coating and an increase within the open circuit voltage (Voc) and fill factor (FF).

**Figure 8:**
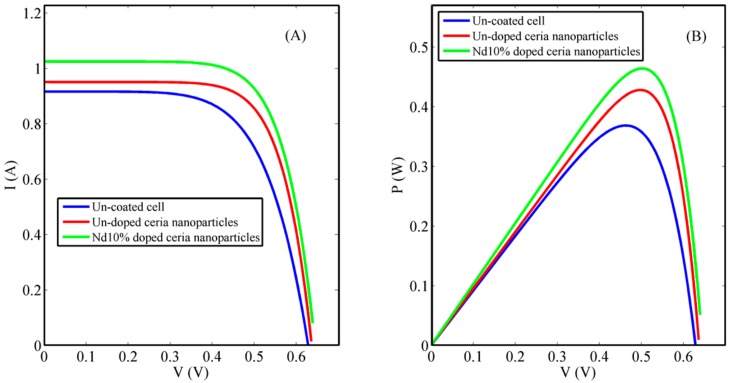
Coated solar cell characteristic curves: (**A**) I-V curve and (**B**) P-V curve.

As discussed in the previous sections, coating the rear surface of Si solar cells with lanthanide-doped ceria nanoparticles may lead to the improvement of both conductivity and optical UV photon absorption via solar cells. This improvement will lead to higher solar cell efficiencies. Spin coating Nd-doped ceria nanoparticles showed enhanced behavior of the I-V curve of the Si solar cells compared to that of un-doped ones, as shown in Figure 8, and illustrated in detail in Table 3. This is due to the improvement within the optical and electrical properties of lanthanide-doped ceria nanoparticles, which were discussed in the previous sections. Generally, this enhancement proves that the impact of increasing the generated photoelectrons is through optical down conversion and better mobility due to a conductive nanostructure coating.

## 4. Discussion

In Figure 1, comparing both Nd-doped and un-doped ceria nanoparticles, it is obvious that lanthanide Nd-dopants shifted the absorbance curves towards higher wavelengths, which corresponds to a reduction of the allowed bandgap values near approximately 3 eV. This reduction indicates that there are more Ce^3+^ ion reductions with correspondingly more O-vacancy formations [17,18].

From Figure 2, it is obvious that un-doped ceria nanoparticles with a 4 mg/mL concentration show the highest photoluminescence intensity among the rest of the concentrations. Un-doped ceria nanoparticles with concentrations higher than 4 mg/mL show fewer photoluminescence intensity peaks as a result of the quenching mechanism. This quenching mechanism might occur due to the excess Ce^3+^ ions within the measured samples that might prevent the excitation photons from reaching the rest of the ions.

Figure 3 shows that the selected lanthanide dopant, Nd, resulted in an increase in the formed O-vacancy concentration due to the increase in the concentration of Ce^3+^ states and with a relative wide band of misaligned trap energy levels due to the formed oxygen vacancies defects. Both optical down-conversion along with improved conductivity through a hopping mechanism through O-vacancies cause the improvement of solar cell efficiency, especially the short-circuit current, as shown in both Figure 7 and Figure 8. Therefore, the improvement of solar cell efficiency has two contributions: optical and electrical. Nd-doped ceria has an improved 5d-4f transition probability, which corresponds to higher fluorescence emission intensity under near violet excitation. That enhances the efficiency of the solar cell through a better utilization of the unabsorbed spectrum around the violet/UV range [9,23,24,25]. The electrical enhancement is correlated to a better formed O-vacancy concentration with less activation energy between cerium ions and the vacancies. That leads to an easier hopping for the generated photoelectrons of the solar cell, with a better conductivity and enhanced short circuit current.

## 5. Conclusions

This paper introduces a promising study of using lanthanide-doped ceria nanoparticles as a backside coating layer on the rear side of the silicon solar cells. The presented work shows full optical and electrical characterization of the synthesized nanoparticles. Experimental results show the visible photoluminescence emitted under near UV excitation and the bandgap values of both un-doped and Nd-doped ceria nanoparticles. These results confirm the existence of the formed O-vacancies associated with the formation of reduced Ce^3+^ ions. These formed O-vacancies could increase the conductivity for any photo-generated electrons in the host nanoparticles through a hopping mechanisms. After spin coating, both un-doped and lanthanide-doped ceria nanoparticles on the back side of Si solar cells show a promising improvement in the solar cell efficiency due to the mutual impact of improved electric conductivity and optical down conversion mechanisms. Doped ceria nanoparticles with 10 wt. % of Nd was the best concentration and resulted in the highest overall efficiency improvement from 14.74% to 18.56%.

## Figures and Tables

**Table 1:** Conductivity of un-doped and Nd-doped ceria nanoparticles.

Condition	Conductivity (µS/cm)
Un-doped ceria nanoparticles	232
Nd 5 wt. % doped ceria nanoparticles	260.7
Nd 10 wt. % doped ceria nanoparticles	270.9

**Table 2:** Comparison between un-coated and un-doped ceria nanoparticle-coated Si solar cell electrical parameters. I_SC_ = short circuit current; V_OC_ = open circuit voltage.

Condition	Concentration (mg/mL)	V_OC_ (V)	I_SC_ (A)	Efficiency (η%)
Un-coated solar cell	0	0.6320	0.9165	14.74
Ceria nanoparticle-coated solar cell	1	0.6313	0.9321	15.72
	4	0.6359	0.9195	17.64
	6	0.6199	0.9510	17.12

**Table 3:** Comparison between ceria nanoparticle- and Nd 10% solution-coated Si solar cell electrical parameters.

Condition	V_OC_ (V)	I_SC_ (A)	Efficiency (η%)
Un-coated solar cell	0.6320	0.9165	14.74
Ceria nanoparticle coated cells	0.6359	0.9195	17.64
Nd 10% ceria nanoparticle coated cells	0.6393	1.0249	18.56
